# Effects of drought duration on terpene profiles, physiological responses, and terpene-related gene expression in rosemary

**DOI:** 10.1186/s12870-026-08193-7

**Published:** 2026-02-16

**Authors:** Doaa Bahaa Eldin Darwish, Mohammed Ali, Fathia A. Soudy, Elsayed Elazazi, Aesha H. Abdel Kawy, Rania M. Makki, Maha Aljabri, Nadiah Al-Sulami, Naeema A. Yahya, Muhammad Zayed

**Affiliations:** 1 Botany Department, Faculty of Science, Mansoura University, Mansoura, 35516 Egypt; 2 Maryout Research Station, Genetic Resources Department, Desert Research Center, 1 Mathaf El-Matarya St., El-Matareya, Cairo, 11753 Egypt; 3 Genetic and Cytology Unit, Genetic Resources Department, Desert Research Center, 1 Mathaf El-Matarya St., El-Matareya, Cairo, 11753 Egypt; 4 Genetics and Genetic Engineering Department, Faculty of Agriculture, Benha University, Moshtohor, 13736 Egypt; 5 Ecophysiology Unit, Plant Ecology and Range Management Department, Desert Research Center, 1 Mathaf El-Matarya St., El-Naam, Cairo, 11753 Egypt; 6 Genetic Resources Section, Agriculture Research Department, Ministry of Municipality, P.O. Box 2727, Doha, Qatar; 7 Department of Biological Sciences, Faculty of Science, King Abdulaziz University (KAU), P.O. Box 80141, Jeddah, 21589 Saudi Arabia; 8 Department of Biology, Faculty of Science, Umm Al-Qura University, Makkah, 21955 Saudi Arabia; 9 Agricultural Research Department, Ministry of Municipality, P.O. Box 2727, Doha, Qatar; 10 Department of Botany and Microbiology, Faculty of Science, Menoufia University, Shebin El-Kom, 32511 Egypt

**Keywords:** Rosemary, Terpenoid biosynthesis, Chromatograms/mass spectra, Lamiacea, Metabolic analysis, Quantitative RT-PCR, Secondary metabolism, Water deficit

## Abstract

Drought is a major environmental constraint limiting plant growth and productivity. This study investigates the effects of drought on rosemary (*Salvia rosmarinus* Spenn.) plantlets subjected to 5, 10, and 15 days of irrigation withholding, with control plants watered regularly every five days. Growth, physiological parameters, and antioxidant enzyme activities were examined. The results revealed reductions in chlorophyll content. Antioxidant enzyme activities—including catalase (CAT), superoxide dismutase (SOD), polyphenol oxidase (PPO), and secondary soluble peroxidase (SPO)—increased, whereas phenylalanine ammonia-lyase (PAL) and ascorbate peroxidase (APX) decreased. Furthermore, the types and quantities of terpenes and other phytochemical compounds produced by rosemary plantlets under drought stress at different time points were analyzed using GC–MS. A total of 710 phytochemical compounds were identified across the following samples: control 5 days, control 10 days, control 15 days, drought 5 days, drought 10 days, and drought 15 days. Overall, the total percentage of monoterpenes decreased under drought, whereas sesquiterpenes, diterpenes, and triterpenes increased. Additionally, RT-qPCR was used to quantify the expression of twelve terpene biosynthesis genes (*SrBDH*,* SrGPS*,* SrFPPS*,* SrGGPP*,* SrCINS1*,* SrCINS2*,* SrTPS-Pin*,* SrHUMS*,* SrKSL2*,* SrFS2*,* SrCPS1*, and *SrTPS1*) under drought conditions relative to the control, to elucidate the relationship between gene expression and terpene type and abundance. In summary, this study highlights the impact of drought on the composition of terpenes in *S. rosmarinus* plantlets, providing valuable insights that may be leveraged to enhance rosemary nutritional value and productivity under drought stress.

**Supplementary Information:**

The online version contains supplementary material available at 10.1186/s12870-026-08193-7.

## Introduction

Biotic and abiotic stressors represent major environmental constraints in Mediterranean countries, primarily driven by drastic reductions in total rainfall [1–3]. These environmental challenges significantly affect the development, growth, and metabolism of medicinal and aromatic plants. Both biotic and abiotic stressors intensify damage, leading to reduced growth, metabolic yield, and biomass production [4].

Recent evidence indicates that flash droughts can rapidly intensify vegetation loss and substantially delay ecosystem recovery, underscoring the urgent need to better understand plant responses to short-term yet increasingly severe water-deficit episodes [5]. In parallel, improving water productivity in arid and semi-arid irrigated systems increasingly depends on mechanistic insights into soil–plant water relations and irrigation optimization strategies supported by process-based datasets, including water isotope approaches [6]. Beyond their effects on growth and biomass, drought conditions can markedly alter the quantity and composition of specialized metabolites that determine the medicinal and economic value of many plant species. Numerous bioactive compounds with analgesic and cardioprotective properties continue to be identified from diverse botanical sources [7, 8], and novel triterpenoid structures are still being reported [9]. At the molecular level, these metabolic outputs are tightly regulated through transcriptional control, such as methyl jasmonate (MeJA)-responsive MYB transcription factors governing triterpenoid biosynthetic pathways, as well as through developmental programs including WOX-mediated root development that directly influences water uptake and drought adaptation [10, 11].

Rosemary *Salvia rosmarinus* Spenn. (syn. *Rosmarinus officinalis* L.) is a well-known ornamental, medicinal, and culinary plant. It belongs to the family Lamiaceae and is distributed across several countries, including Egypt, Albania, Tunisia, Algeria, Libya, Morocco, Turkey, the Balearic Islands, Corsica, Spain, Cyprus, the East Aegean Islands, France, Greece, Italy, and other Mediterranean regions [3]. Its tissues contain high concentrations of volatile (essential) oils rich in terpene derivatives such as α-pinene, cis-α-terpineol, (+)-camphor, β-caryophyllene, levo-β-pinene, germacrene-A, thujone, phytane, ledol, squalene, farnesane, 1,8-cineole, and (+)-phytol [3, 4]. These compounds are recognized for their anti-inflammatory, antibacterial, lubricant, antitumor, antiseptic, spasmolytic, antioxidant, analgesic, cardiovascular, anti-cholinesterase, and antidiabetic [1–3, 12–20].

Globally, approximately 1,000 *Salvia* species have been recorded (e.g., *S. rosmarinus*, *S. tuxtlensis*, *S. aegyptiaca*, *S. japonica*, *S. aethiopis*, *S. acerifolia*, *S. aureus*, *S. santolinifolia*, *S. acuminata*, *S. argentea*, *S. hydrangea*, *S. tomentosa*, *S. africana*, *S. miltiorrhiza*, *S. glabrescens*, *S. arrabidae*, *S. amplifrons*, *S. nipponica*, *S. chloroleuca*, and *S. algeriensis*), all listed in the Plant List database of the World Flora Online (WFO) [3, 12, 13, 15–21].

Terpenes constitute a major class of plant secondary metabolites, comprising over 60,000 known structures (Khater 2022). All terpenes originate from five-carbon precursors synthesized through either the mevalonic acid (MVA) pathway or the methyl-D-erythritol phosphate (MEP) pathway. These C5 units are subsequently polymerized to form geranyl diphosphate (GPP) and farnesyl diphosphate (FPP), which undergo cyclization, rearrangement, and additional modifications [13, 15]. A wide variety of terpene classes—including monoterpenes (C₁₀H₁₆), sesquiterpenes (C₁₅H₂₄), diterpenes (C₂₀H₃₂), sesterterpenes (C₂₅H₄₀), triterpenes (C₃₀H₄₈), sesquarterpenes (C₃₅H₅₆), and tetraterpenes (C₄₀H₆₄)—are synthesized by terpene synthase (TPS) enzymes using GPP, FPP, and geranylgeranyl diphosphate (GGPP) as substrates [21–25].

Numerous full-length and partial cDNAs encoding mono-, sesqui-, di-, sester-, tri-, and tetraterpene synthases have been characterized from Lamiaceae plants, particularly the genus *Salvia* [25–29]. These terpene synthases contain conserved motifs in their C-terminal and N-terminal domains that determine product specificity [3, 22, 27, 30–33].

Many studies have demonstrated that drought frequently up-regulates terpene biosynthesis genes, resulting in increased terpene production. These terpenes may contribute to drought tolerance by scavenging reactive oxygen species (ROS) and protecting plant tissues from oxidative damage. However, terpene synthase gene regulation varies depending on species and drought severity, with some genes being up- or down-regulated [34, 35]. Sakthi et al. (2025) [36] reviewed that the modulation of terpene profiles triggered by drought emphasizes their potential roles in enhancing plant adaptive capacity, which is crucial for coping with the escalating risks of climate change. Additionally, rosemary has been suggested for cultivation in marginal soils, as its growth is only marginally affected by drought stress, while drought enhances terpene production in its essential oils, potentially increasing their medicinal properties and commercial value [37]. Thus, drought can be viewed as an opportunity rather than a challenge when suitable plants are selected and compounds of interest, whose production increases under drought conditions, are utilized, given their societal, medical, and commercial importance. Therefore, this study aims to: (i) elucidate the genetic and physiological responses of *S. rosmarinus* plantlets exposed to different drought durations; (ii) examine the effects of drought duration on antioxidant enzyme activities; (iii) evaluate changes in metabolic profiles under drought stress; (iv) investigate the relationship between terpenes and drought severity; (v) quantify the expression of key terpene biosynthesis genes under varying drought conditions using quantitative reverse transcription PCR (RT-qPCR); and (vi) integrate physiological, metabolic, and qPCR data to elucidate the regulatory mechanisms underlying terpene accumulation patterns in response to drought stress.

## Materials and methods

### Plant materials, growth conditions, treatments, and sampling

*Salvia rosmarinus* plantlets were obtained from the Maryout Research Station, Desert Research Centre (DRC), Alexandria Governorate, Egypt. The plantlets were kindly provided by Prof. Dr. Adel Abdel Wahed, former head of the Maryout Research Station. In this study, we used the soil pot water control method as described by Wang et al. (2024) [38], in which individual plantlets were grown in black plastic pots (10 × 25 cm) containing 1.5 kg of a 1:1 clay–sand mixture from a single homogenized batch to reduce variability in soil microbial communities, and then transferred to a naturally ventilated open greenhouse where climatic conditions, including temperature, relative humidity, and solar radiation, were not controlled and closely followed ambient environmental conditions. Moreover, to minimize positional effects, pots were randomly arranged within the phytotron throughout the experiment, and plantlets were irrigated every five days with tap water and supplemented with NPK fertilizer for 14 days. In addition, pots were maintained at fixed randomized locations throughout the experiment to minimize positional effects, and plantlets were irrigated every five days with tap water and supplemented with NPK fertilizer (3 g/plant ammonium sulphate (20.6% N), 2/pot of calcium superphosphate (15.5% P_2_O_5_) and 1 g/plant potassium sulphate (48% K_2_O)) for 14 days [39]. Furthermore, before drought treatment, pots containing rosemary plantlets regularly were watered with tap water at field capacity (FC). For determination the FC level of the soil, pots containing 1.5 kg of dry clay–sand mixture in a ratio of 1:1 (v/v) were weighed (W1). These pots were watered to saturation and excess water flows under gravity. Pots were covered by plastic bags to prevent evaporation and after 48 h pots were weighed (W2). The difference between the two weights (W2-W1) was the amount of soil saturation point (100% FC). For the determination of irrigation volumes, following formulae were used [38, 40, 41]:

$$50\%FC=0.5\times(W2-W1)$$

$$25\%FC=0.25\times(W2-W1)$$

Drought treatments were then initiated through a time-based water-withholding irrigation regime. Irrigation was withheld for 5 days (100% FC), 10 days (50% FC), and 15 days (25% FC) to increase drought severity, while control plantlets (wild type) were watered with tap water every five days throughout the experiment. For biochemical analysis, terpene profiling, antioxidant enzyme activity, and gene-expression studies, samples were collected from both control and drought-treated plantlets at the same time. Each sample consisted of three biological replicates.

The collected samples were designated as follows:

- 5 days control (5DC),
- 10 days control (10DC),
- 15 days control (15DC),
- 5 days drought (5DDS),
- 10 days drought (10DDS),
- 15 days drought (15DDS).

These abbreviations refer to wild-type and drought-treated plantlets sampled after 5, 10, and 15 days. To minimize circadian rhythm–related variations, all samples were collected simultaneously on the same day at the same time and stored at − 20 °C for short time until further analyses.

### Isolation of phytochemical compounds using hexane

GC–MS was used to compare terpenoid profiles between wild-type and drought-treated plantlets. For each treatment, three independent biological replicates (one plantlet per replicate) were analyzed. Twenty-four leaves from each group (eight leaves per plantlet) were pooled and prepared for terpene extraction following previously described protocols [4, 16, 17, 20, 27, 37, 42, 43]. The resulting solvent extract was transferred using a glass pipette into 10-ml glass centrifuge tubes with screw-cap vials containing silicone/PTFE septa and centrifuged at 5,100 rpm for 9 min at 4 °C to remove plant debris. A 1µL aliquot from each biological replicate was injected into a Shimadzu GCMS-QP2010 Ultra system for analysis. Terpenoids were identified using the Wiley GC/MS Library (10th Edition), VOC Analysis Software, and the NIST Library (2014 Edition) [4, 16].

### GC-MS analysis of hexane extracts

GC analysis was performed using a Shimadzu model GCMS-QP2010 Ultra (Tokyo, Japan) system. An approximately 1 µl aliquot from each biological replicate was injected (split ratios of 15:1) into a GC-MS equipped with an HP-5 fused silica capillary column (30 m × 0.25 mm ID, 0.25 μm film thickness). Helium was used as the carrier gas at a constant flow of 1.0 mL min^**− 1**^. The mass spectra were monitored between 50 and 450 m/z. Temperature was initially under isothermal conditions at 60 °C for 10 min. Temperature was then increased at a rate of 4 °C min^**− 1**^ to 220 °C, held isothermal at 220 °C for 10 min, increased by 1 °C min^**− 1**^ to 240 °C, held isothermal at 240 °C for 2 min, and finally held isothermal for 10 min at 350 °C. The identification of the volatile constituents were done by parallel comparison of their recorded mass spectra with the data stored in the Wiley GC/MS Library (10th Edition) (Wiley, New York, NY, USA), and the retention time index (http://massfinder.com/wiki/MassFinder_Analysing_your_own_data), with the Volatile Organic Compounds (VOC) Analysis S/W software, and the NIST Library (2014 edition), The Adams Library (http://essentialoilcomponentsbygcms.com/list-of-compounds-in-the-essential-oil-components-database/), and the Terpenoids Library (http://massfinder.com/wiki/Terpenoids_Library_List). The relative% amount of each component was calculated by comparing its average peak area to the total areas, as well as Retention time index. (All of the experiments were performed simultaneously three times under the same conditions for each isolation technique with total GC running time was 80 min [4, 16].

### Quantification of terpene-related gene expression under drought stress using qRT-PCR

To validate the expression of terpene biosynthetic genes in *S. rosmarinus* under drought stress, twelve genes were selected based on our previous studies [12, 17, 20], which demonstrated their correlation with terpene metabolism in rosemary. The terpene biosynthetic genes selected for analysis in this study, together with their corresponding nucleotide sequences retrieved from the GenBank database [44–46], included (+)-borneol dehydrogenase (*SrBDH*, MT857224.1), geranyl diphosphate synthase (*SrGPS*, KY399788), farnesyl pyrophosphate synthase (*SrFPPS*, KY399787), geranylgeranyl pyrophosphate synthase (*SrGGPP*, KY486794), cineole synthase 1 (*SrCINS1*, JX050194.1), 1,8-cineole synthase (*SrCINS2*, KX893964), pinene synthase (*SrTPS-Pin*, EF495245.1), α-humulene/β-caryophyllene synthase (*SrHUMS*, KX893973), kaurene synthase-like 2 (*SrKSL2*, KF805859.1), ferruginol synthase (*SrFS2*, KP091844.1), copalyl diphosphate synthase (*SrCPS1*, KF805857.1), and limonene synthase (*SrTPS1*, DQ421800.1). β-Actin (*SrBACTIN*, HM231319.1) was used as the internal reference gene for qPCR expression analysis (Table S1).

Total RNA was extracted immediately from the leaves of control and drought-treated plantlets at the three time points. First-strand cDNA synthesis was performed using reverse transcriptase master mix and none-reverse transcriptase reactions. For each reaction, 6 µL of the appropriate master mix was combined with 14 µL of template RNA and incubated at 42 °C for 30 min. The reaction was terminated at 95 °C for 3 min and immediately chilled on ice. The synthesized cDNA was then tenfold diluted, and Quantiscript SYBR Green PCR Master Mix was prepared according to manufacturer’s instructions. Finally, The Real-time PCR program was performed on a CFX96 Dx Real-Time PCR Detection System using three biological replicates, and consisted of an initial denaturation (95 °C/3 min), followed by 40 amplification cycles of denaturation (95 °C/10 s), annealing at either (58–60 °C/30 s), and extension (72 °C/20 s), with a final extension step at 65 °C for 1 min [4, 12, 17, 18, 20, 42, 47–49]. *SrACTIN* was used as the reference gene. All primers were designed using the IDT DNA database, and their sequences are provided in Table S1 [12, 17, 20]. qRT-PCR Relative expression levels were calculated using the reference gene *SrACTIN* and the 2^−∆∆Ct^ method.

### Measurement of antioxidant enzyme activities and physiological/biochemical indices

Activities of antioxidant enzymes were determined following the protocols of Eggink et al. (2001), El-Mahdy et al. (2024), and Abbas et al. (2024) [43, 50, 51]. Fresh leaf tissue (50 mg) from control and drought-stressed plantlets was ground to a fine powder on ice with a buffer (like phosphate buffer with EDTA) to prevent oxidation, then centrifuging to get the liquid extract (supernatant) containing enzymes like and processed according to the specific extraction methods for catalase (CAT), phenylalanine ammonia-lyase (PAL), ascorbate peroxidase (APX), superoxide dismutase (SOD), polyphenol oxidase (PPO), and soluble peroxidase (SPO). After that the activity of each antioxidant enzyme was assay using a spectrophotometer. Moreover, Protein content was determined according to Lowry et al. (1951) [52], and enzyme activities were normalized to the same protein concentration across all drought duration treatments.

For chlorophyll a, chlorophyll b, and total chlorophyll (a + b), 60 mg of fresh tissue was homogenized in 5 mL of 95% ethanol, following El-Mahdy et al. (2024) and Ali et al. (2025) [49, 50]. The extract was heated at 65–70 °C for 32 min. Optical densities (ODs) were measured at 664.2 nm and 648.6 nm using a JENWAY 6505 UV/VIS spectrophotometer. Three biological replicates were used for each treatment.

Chlorophyll contents were calculated as follows:

- Chl a (mg/g FW) = (13.36 × A664.2) – (5.19 × A648.6).
- Chl b (mg/g FW) = (27.43 × A648.6) – (8.12 × A664.2).
- Total Chl (a + b) = Chl a + Chl b.

### Statistical analysis

Analysis of variance (ANOVA) was performed to compare mean values of antioxidant enzyme activities and physiological and biochemical parameters in wild-type and drought-stressed *S. rosmarinus* plantlets. The experiment was conducted using a completely randomized design (CRD) with three replicates. Variations in physiological parameters (total chlorophyll, chlorophyll a, and chlorophyll b) and antioxidant enzyme activities (CAT, PAL, APX, SOD, PPO, and SPO) across drought durations were analyzed. Statistical analyses were performed using SPSS version 21.0 [51].

## Results

### Morphological changes of *S. rosmarinus* under various drought times

**Fig. 1:**
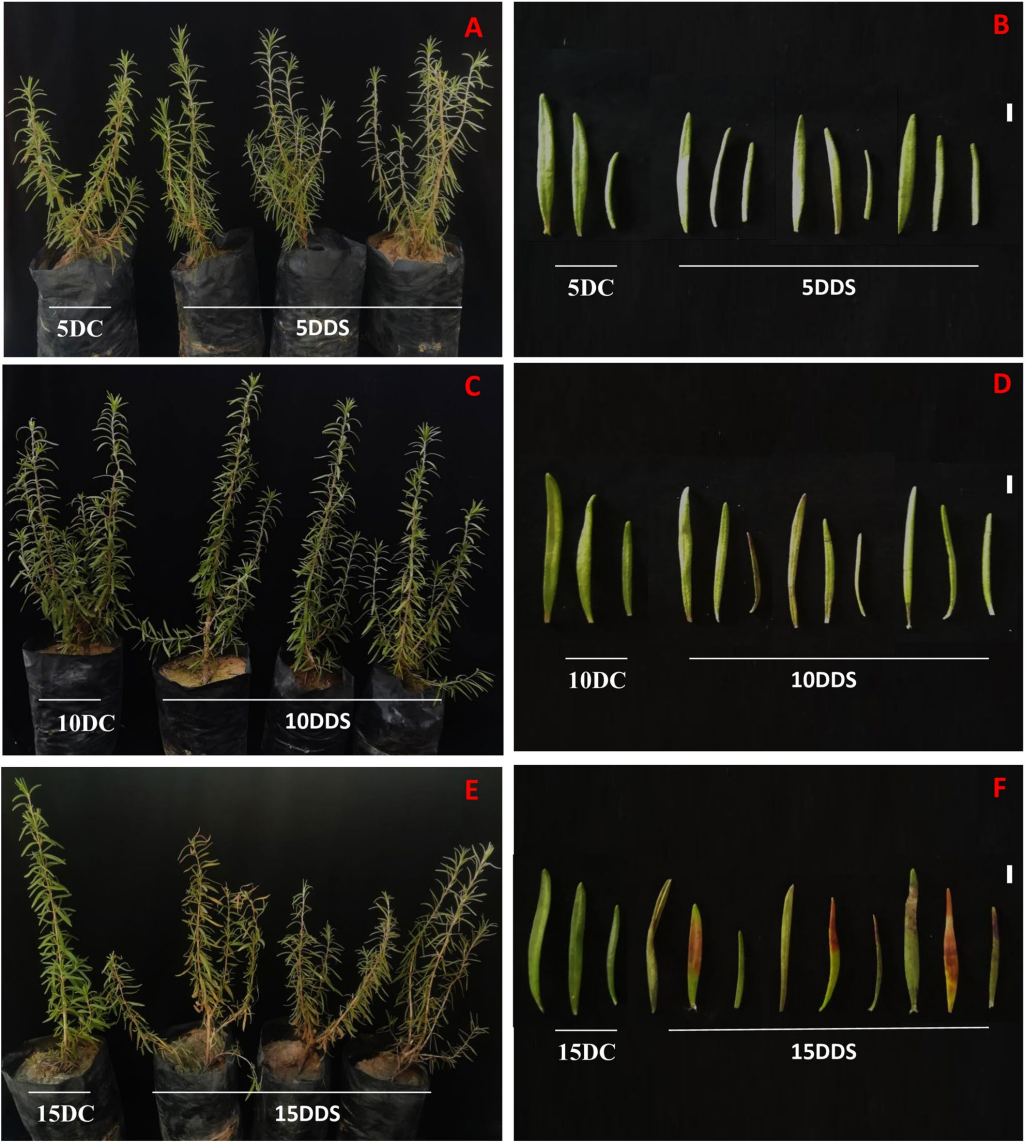
Effect of drought stressors at different times on the growth and development of *S. rosmarinus* plantlets. **A**, **C** and **E** *S. rosmarinus* plantlets at 5, 10 and 15 days without drought treatment ((5 days control (5DC), 10 days control (10DC), 15 days control (15DC)) and under treatment with drought (5 days drought (5DDS), 10 days drought (10DDS) and 15 days drought (15DDS)). **B**, **D**, **F** Leave phenotype of *S. rosmarinus* plantlets at 5, 10 and 15 days without drought treatment (5DC, 10DC and 15DC) and under treatment with drought (5DC, 10DC, 15DC). Scale bar = 1 cm

Drought strongly influences all the aspects of medicinal plant’s life, particularly *S. rosmarinus* plantlets, resulting in many morphological changes in the growth rate, shape and colour of the leaves. So in this experiment we assess the behaviour of *S. rosmarinus* plantlets under various drought times. And from our results, we found the plantlet morphologically specially the leaf form and colour has been affected by different times of drought stressors at different development stages (Fig. 1).

### Identification of terpenoid and chemical composition from the hexane extracts of S. rosmarinus plantlets under different drought times by GC-MS

**Fig. 2:**
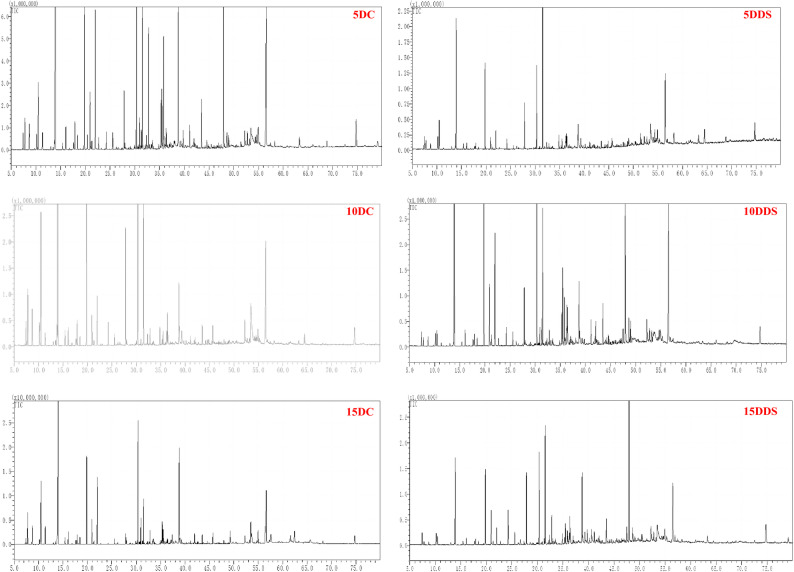
Typical GC-MS mass spectragraphs for terpenoids from hexane extracts of *S. rosmarinus* plantlet at 5, 10 and 15 days without drought treatment (5DC, 10DC and 15DC) and under treatment with drought (5DDS, 10DDS and 15DDS)

**Fig. 3:**
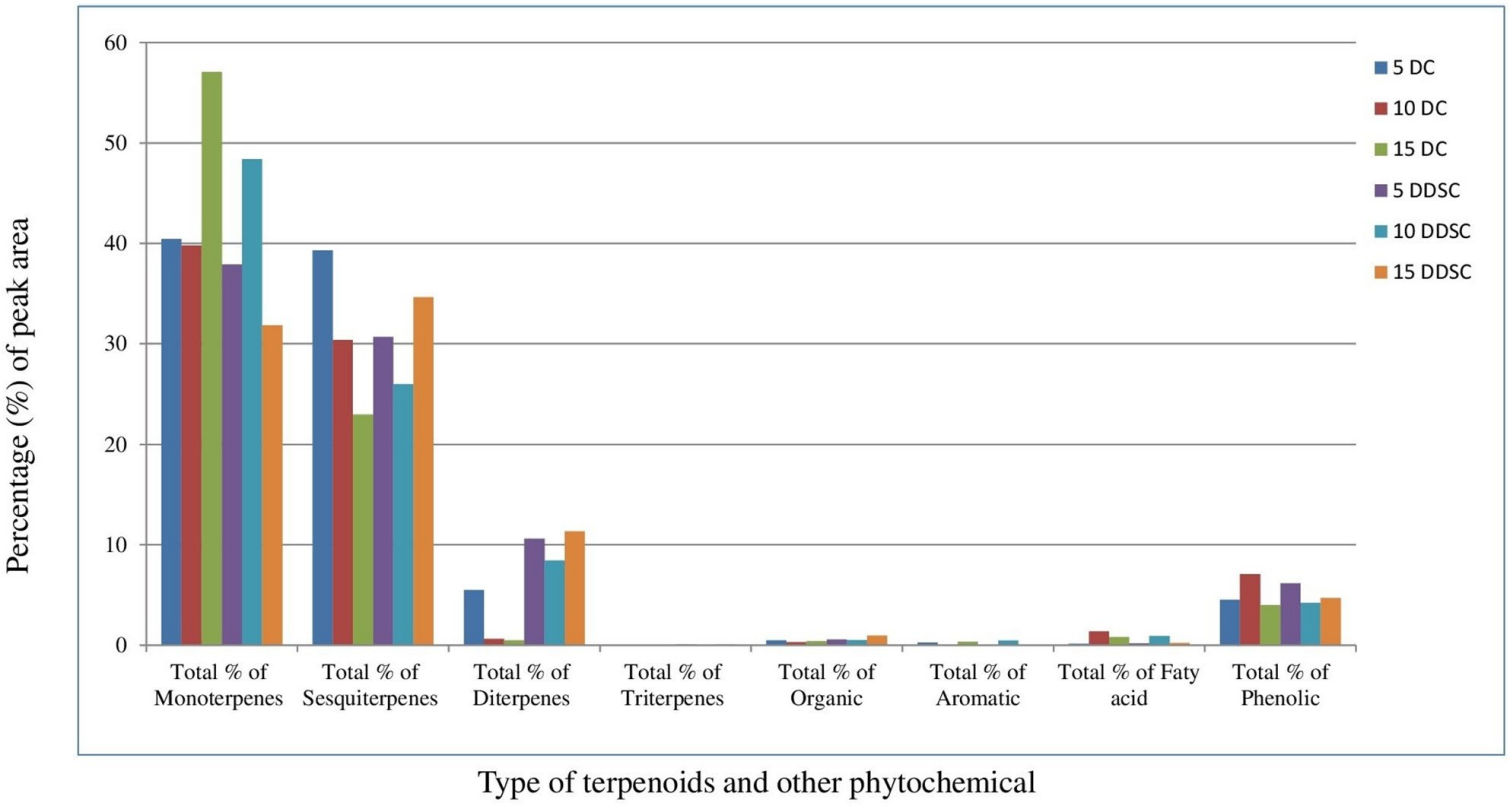
The total percentage of all terpenoid types and other phytochemical from hexane extracts of *S. rosmarinus* plantlet at 5, 10 and 15 days without drought treatment (5DC, 10DC and 15DC) and under treatment with drought (5DDS, 10DDS and 15DDS)

The type and quantity of various terpenoid compounds from the hexane extracts of *S. rosmarinus* plantlets under different drought times were determined by GC-MS, as shown in Figs. 2 and 3, and Table 1 and Table S2. *S. rosmarinus* plantlets after being treated with different drought times produced various types and quantities of mono-, sesquit-, dit-and triterpenes when compared with the control. The numbers of obtained terpenoid and other phytochemical compounds from *S. rosmarinus* plantlets under different treatments (5DC, 10DC, 15DC, 5DDS, 10DDS and 15DDS) were 152 (100%), 83 (100%), 189 (99.91%), 84 (100%), 106 (98.92%) and 96 (99.96%), respectively. From the GC-MS analysis, we identified 710 phytochemical compounds using hexane extracts from the six samples representing the *S. rosmarinus* plantlets after treated with different drought times and control. In *S. rosmarinus* plantlets after 5 days without drought treatment (5DC), the monoterpene compounds were shown as the main group (40.45%), followed by the group of sesquiterpene compounds (39.31%), diterpene compounds (5.49%), phenolic compounds (4.53%), organic compounds (0.49%), aromatic compounds (0.26%) and fatty acid compounds (0.15%). After 10 days without drought treatment (10DC), the monoterpene compounds were shown as the main group (39.79%), followed by the group of sesquiterpene compounds (30.38%), phenolic compounds (7.08%), Fatty acid compounds (1.37%), diterpene compounds (0.62%) and organic compounds (0.3%). Moreover, in *S. rosmarinus* plantlets after 15 days without drought treatment (15DC), the monoterpene compounds were shown as the main group (57.08%), followed by the group of sesquiterpene compounds (22.95%), phenolic compounds (4.02%), fatty acid compounds (0.82%), diterpene compounds (0.5%), organic compounds (0.42%) and aromatic compounds (0.35%). On the other side, in *S. rosmarinus* plantlets after 5 days from drought treatment (5DDS), the monoterpene compounds were shown as the main group (37.91%), followed by the group of sesquiterpene compounds (30.67%), diterpene compounds (10.6%), phenolic compounds (6.18%), organic compounds (0.58%), fatty acid compounds (0.19%) and triterpene compound (0.06%).

In addation, in *S. rosmarinus* plantlets after 10 days from drought treatment (10DDS), the monoterpene compounds were shown as the main group (48.4%), followed by the group of sesquiterpene compounds (25.98%), diterpene compounds (8.43%), Phenolic compounds (4.23%), Faty acid compounds (0.91%), organic compounds (0.52%), Aromatic compounds (0.47%) and triterpene compound (0.01%).While, in *S. rosmarinus* plantlets after 15 days from drought treatment (15DDS), the sesquiterpene compounds were shown as the main group (34.64%), followed by the group of monoterpene compounds (31.85%), diterpene compounds (11.34%), Phenolic compounds (4.7%), Organic compounds (0.94%), Fatty acid compounds (0.21%) and triterpene compound (0.06%) (Figs. 2 and 3; Table 1 and Table S2).

**Table 1:** The list of major terpenoid and phytochemical composition of the hexane extracts of *S. rosmarinus* under different drought times

No.	Compound Name	RT	Formula	MW/Da	Terpene Type	Control			Drought Durations		
						5 DC	10 DC	15 DC	5 DDS	10 DDS	15 DDS
1	Cyclene	7.235	C10H16	136.234	Orga			0.05			
2	Alpha-Phellandrene	7.406	C10H16	136.234	Mono	0.51	0.94	0.43	1.2	0.59	0.89
3	alpha-Pinene	7.764	C10H16	136.234	Mono	0.94	2.19	2.24	0.8	0.36	0.21
4	Camphene	8.669	C10H16	136.234	Mono	0.76	1.45	1.31	0.47	0.39	0.25
5	Artificial Almond Oil	9.495	C7H6O	106.1219	organic			0.01			
6	cis-sabinene	10.161	C10H16	136.234	Mono	0.48	0.89	0.33	1.22	0.54	0.87
7	β-Pinene	10.45	C10H16	136.234	Mono	2.02	5.31	4.49	2.86	0.65	0.64
8	β-Myrcene	11.329	C10H16	136.234	Mono	0.51	0.49	1.25	0.15	0.14	0.04
9	(-)-Alpha-Phellandrene	12.357	C10H16	136.234	Mono				0.04		
10	(R)-(-)-α-Phellandrene	12.349	C10H16	136.234	Mono	0.01	0.14	0.02	0.27	0.12	0.17
11	p-Mentha-1,4(8)-diene	13.009	C10H16	136.234	Mono	0.09		0.11			
12	o-Cymene	13.479	C10H14	134.2182	Aromatic	0.07	0.17	0.21			
13	D-Limonene	13.745	C10H16	136.234	Mono	0.29	0.84			0.15	
14	Eucalyptol	13.941	C10H18O	154.2493	Mono	17.74	23.14	20.97	12.82	12.45	6.65
15	β-Ocimene	14.832	C10H16	136.234	Mono			0.01			
16	1,4-p-Menthadiene	15.406	C10H16	136.234	Mono	0.19	0.59	0.48	0.47	0.17	0.24
17	trans-β-Terpineol	16.055	C10H18O	154.2493	Mono	0.68	0.69	0.88	0.54	0.68	0.5
18	(Z)-β-Terpinolene	16.813	C10H16	136.234	Mono	0.04	0.11	0.08	0.11	0.05	0.06
19	iso-β-terpineol	17.635	C10H16	136.234	Mono	0.21	0.26	0.28		0.27	
20	δ-Thujone	17.892	C10H16O	152.2334	Mono	0.83	0.97	0.67	0.55	0.5	0.4
21	Phenylethyl Alcohol	18.184	C8H10O	122.1644	organic			0.08			
22	iso-3-Thujone	18.454	C10H16O	152.2334	Mono	0.43	0.35	0.52	0.18	0.34	0.25
23	D-(+)-Camphor	19.809	C10H16O	152.2334	Mono	4.82	9.46	6.21	8.38	7.37	5.72
24	trans-Pinocamphone	20.412	C10H16O	152.2334	Mono	0.42				0.03	2.59
25	L-α-Terpineol	20.919	C10H18O	154.2493	Mono	1.72	1.18	1.74	1.12	2.62	
26	(Z)-Pinocamphone	21.112	C10H16O	152.2334	Mono	0.21					
27	1-para-Menthen-4-ol	21.331	C10H18O	154.2493	Mono	0.24	0.17	0.25		0.43	0.25
28	.alpha.-Terpineol	22.002	C10H18O	154.2493	Mono	4.1	1.92	4.69	1.81	4.73	1.26
29	Pinanediol	22.702	C10H18O2	170.2487	Mono	0.35	0.06	0.09	0.13	0.31	0.25
30	2,3-Pinanediol	23.3	C10H18O2	170.2487	Mono	0.04				0.06	
31	Linolool, formate	24.24	C11H18O2	182.2594	Mono	0.51	0.88	0.06	0.96	0.78	2.57
32	Isobornyl acetate	25.544	C12H20O2	196.286	Orgain	0.49	0.42	0.38	0.3	0.58	0.94
33	Carvacrol	26.138	C10H14O	150.2176	Mono			0.02			
34	γ-Elemene	27.273	C15H24	204.3511	Sesqui	0.04					
35	3,7-Octadiene-2,6-diol, 2,6-dimethyl-	27.373	C10H18O2	170.2487	Mono						0.25
36	cis-2-acetoxy-1,8-cineole	27.542	C12H20O3	212.2854	Mono	0.04		0.01			
38	p-menth-1-en-8-yl acetate	27.826	C12H20O2	196.286	Mono	1.72	4.49	0.73	4.4	2.44	5.37
39	Eugenol	28.014	C10H12O2	164.2011	aromatic	0.19	0.18	0.26			
40	(Z)−8-Hydroxylinalool	28.596	C10H18O2	170.2487	Mono						0.16
41	α-Cubebene	28.651	C15H24	204.3511	Sesqui			0.08			
42	α-Copaene	28.828	C15H24	204.3511	Sesquit	0.06		0.09			
43	(-)-β-Bourbonene	29.1	C15H24	204.3511	Sesquit	0.09	0.04	0.03	0.08	0.14	
44	Isocaryophyllene	29.818	C15H24	204.3511	Sesquit	0.03			8.08		
45	6-epi-β-Cubebene	29.256	C15H24	204.3511	Sesquit			0.03			
46	9-epi-Caryophyllene	29.82	C15H24	204.3511	Sesquit			0.02			
47	ι-Gurjunene	29.901	C15H24	204.3511	Sesquit	0.12	0.06	0.21			
48	Caryophyllene	30.351	C15H24	204.3511	Sesquit	12.77	7.7	8.62		8.87	6.94
49	Alloaromadendrene	30.575	C15H24	204.3511	Sesquit	0.08	0.22	0.17		0.76	
50	1(10)-Aristolene	30.673	C15H24	204.3511	Sesquit	0.09		0.14			
51	(+)-γ-Gurjunene	30.803	C15H24	204.3511	Sesquit	0.12		0.23			
52	α-Aromadendrene	30.942	C15H24	204.3511	Sesquit	0.92		1.83	0.24		0.17
53	γ-Gurjunene	31.07	C15H24	204.3511	Sesquit			0.02			
54	Sativene, (+)-	31.196	C15H24	204.3511	Sesquit	0.09		0.22			
55	α-Gurjunene	31.418	C15H24	204.3511	Sesquit	0.07		0.04			
56	Humulene	31.52	C15H24	204.3511	Sesquit	4.25	9.03	3.17	13.97	5.74	8.85
57	β-Aromadendrene	31.648	C15H24	204.3511	Sesquit	0.11		0.1			
58	1β,4βH,10βH-Guaia-5,11-diene	32.07	C15H24	204.3511	Sesquit			0.05			
59	γ-Muurolene	32.141	C15H24	204.3511	Sesquit	0.11		0.07		0.13	0.23
60	γ-Bulgarene	32.34	C15H24	204.3511	Sesquit	0.39	0.4	0.2	0.59	0.27	0.68
61	α-Selinene	32.596	C15H24	204.3511	Sesquit	0.12		0.16			
62	δ-Amorphene	32.663	C15H24	204.3511	Sesquit	0.06		0.16			
63	Elemene isomer	32.809	C15H24	204.3511	Sesquit	3.57	0.59	0.95	0.43	0.62	2.09
64	.alpha.-Muurolene	32.91	C15H24	204.3511	Sesquit	0.04		0.06			
65	Stavox	33.015	C15H24O	220.3505	Sesquit	0.13		0.05		0.21	0.41
66	δ-muurolene	33.358	C15H24	204.3511	Sesquit	0.15		0.23		0.17	0.18
67	Cadina-1(10),4-diene	33.511	C15H24	204.3511	Sesquit	0.26	0.16	0.4	0.22	0.22	0.23
68	Isocaryophyllene oxide	33.712	C15H24O	220.3505	Sesquit	0.09		0.17			
69	(+)-Ledol; d-Ledol	33.895	C15H26O	222.3663	Sesquit			0.05			
70	Cadine-1,4-diene	33.968	C15H24	204.3511	Sesquit			0.02			
71	epi- α-Muurolene	34.084	C15H24	204.3511	Sesquit			0.03			
72	(+)-Ledol; d-Ledol	34.856	C15H26O	222.3663	Sesquit	0.14	0.69	0.03	1.28	0.21	0.77
73	Epiglobulol	34.871	C15H26O	222.3663	Sesquit			0.16			
74	(-)-Spathulenol	35.32	C15H24O	220.3505	Sesquit	1.39	0.18	1.56		1.31	0.42
75	cis-Caryophyllene epoxide	35.48	C15H24O	220.3505	Sesquit	1.73	0.52	1.04	0.94	3.22	1.51
76	(+)-Ledol;	35.589	C15H26O	222.3663	Sesquit	0.15		0.41		0.14	0.95
77	d-Ledol	35.861	C15H26O	222.3663	Sesquit	3.32		0.2		2.02	
78	(-)-γ-Elemene	35.945	C15H24	204.3511	Sesquit	0.37		0.11		0.3	0.46
79	(-)-Ledol; -	36.17	C15H26O	222.3663	Sesquit	0.06		0.07			
80	Naphthalene, decahydro-, cis-	36.313	C10H18	138.2499	Mono	0.52	0.47	0.3	1.31	1.59	1.95
81	Viridiflorol	36.583	C15H26O	222.3663	Sesquit	0.07		0.05	0.22		
82	trans-caryophyllene oxide	36.985	C15H24O	220.3505	Sesquit	0.12	0.11	0.09		0.32	0.52
83	(+)(-)-caryophyllene oxide	37.112	C15H24O	220.3505	Sesquit	0.15		0.26		0.26	
84	Bicyclo[4.4.0]dec-1-ene, 2-isopropyl-5-methyl-9-methylene-	37.285	C15H24	204.3511	Sesquit	0.03		0.1			
85	10-epi-Elemol	37.354	C15H26O	222.3663	Sesquit	0.1		0.64	0.15	0.13	
86	(+)-Ledol; d-Ledol	37.637	C15H26O	222.3663	Sesquit	0.05		0.16			
87	levo-β-Elemene	37.89	C15H24	204.3511	Sesquit	0.04		0.05			
88	(E)-Caryophyllene	37.962	C15H24	204.3511	Sesqui	0.16		0.14			
89	(-)-Caryophyllene oxide	38.069	C15H24O	220.3505	Sesquit	0.12	0.07		0.22	0.17	0.22
90	(+)-Ledol; d-Ledol	38.112	C15H26O	222.3663	Sesquit			0.21			
91	Guaia-1(10),11-diene; α-Bulnesene; δ-Guaiene;	38.227	C15H24	204.3511	sesqui			0.05			
92	abd-7,13(E)-dien-15-yl acetate	38.801	C22H36O2	332.52	Diter			6.67			
93	Carotol	38.757	C15H26O	222.3663	sesqui	4.96	2.36		2.21	2.55	5.25
94	d-Viridiflorol	39.468	C15H24	204.3511	Sesquit	0.04		0.09			
95	Shyobunone	39.765	C15H24O	220.3505	Sesquit	0.49	0.11	0.05	0.23	0.21	0.96
96	β-Calarene	40.177	C15H24	204.3511	Sesquit	0.08	0.16		0.51		0.92
97	(E)-β-Elemene	40.236	C15H24	204.3511	Sesquit			0.14			
98	β-Gurjunene (calarene)	40.613	C15H24	204.3511	Sesquit	0.05		0.06		0.06	
99	(+)-(E)-Limonene oxide	40.85	C10H16O	152.2334	Mono			0.06			
100	Spathulenol	40.964	C15H24O	220.3505	Sesquit			0.1			
101	(-)-β-Bourbonene	41.121	C15H24	204.3511	Sesquit	0.64	0.3	0.21	0.5	1	0.76
102	Eudesm-11-en-1-ol	41.645	C15H26O	222.3663	Sesquit			0.05			
103	Isocaryophyllene, 5,6-epoxide	41.898	C15H24	204.3511	Sesquit	0.24		0.72		0.31	0.28
104	Nopol (terpene)	44.191	C11H18O	166.26	Mono			0.04			
105	Kaur-15-ene	44.548	C20H32	272.4681	Diter	0.23	0.18	0.18		0.39	0.2
106	Palmitic acid, methyl ester	44.837	C17H34O2	270.4507	Faty acid	0.11	0.14	0.08	0.58	0.19	
107	α-Curcumene	45.458	C15H22	202.3352	Sesquit	0.1	0.13		0.35		0.33
108	Palmitic acid (hexadecanoic acid)	45.681	C16H32O2	256.4241	faty acid		0.68	0.76	0.79		
109	Guaia-1(10),11-diene; α-Bulnesene; δ-Guaiene;	45.928	C15H24	204.3511	sesqui	0.03		0.05		0.13	
110	Ledene	46.021	C15H24	204.3511	sesqui	0.08		0.04			
111	Kaur-16-ene	46.314	C20H32	272.4681	Diter	0.04		0.04			
112	Hexadecanoic acid, ethyl ester	46.518	C18H36O2	284.4772	Faty acid	0.04		0.07			0.21
113	cis-β-Farnesene	46.592	C15H24	204.3511	sesqui	0.08					
114	2-epi-(E)-β-Caryophyllene	46.758	C15H24	204.3511	sesqui	0.08		0.07			
115	trans-β-Caryophyllene oxide	46.929	C15H24	204.3511	Sesquit	0.14	0.12	0.1			
116	Labd-14-ene-8,13-diol, (13R)-	47.04	C20H36O2	308.4986	Diter	0.04		0.03		0.19	
117	Sclareol	47.949	C20H36O2	308.4986	Diter	4.88	0.17	0.23	0.36	9.56	11.07
118	(+)-Isophyllocladene	48.236	C20H32	272.4681	Diter			0.08			
119	(+)-d-Ledol	48.64	C15H26O	222.3663	Sesquit	0.44				1.04	1.03
120	cis-Phytol; trans-Phytol	49.383	C20H40O	296.531	diter			0.9		0.07	
121	(-)-Bisabolol oxide B	49.678	C15H26O2	238.3657	Sesquit			0.06	0.16		0.26
122	p-Menthane, 1,2	49.89	C10H16O2	168.2328	Mono	0.03	0.09	0.13		0.12	
123	Isopinocamphone	50.775	C10H16O	152.2334	Mono					0.03	
124	Eudesm-11-en-1-ol	50.863	C15H26O	222.3663	Sesqui			0.06			
125	1,3a-Ethano(1 H)inden-4-ol, octahydro-2,2,4,7a-tetramethyl-	53.632	C15H26O	222.3663	Sesquit			0.68			
126	Sugiol	54.681	C20H28O2	300.4351	Diter	0.27	0.15	0.16		0.39	
127	8-Methyloctahydrocoumarin	55.402	C10H16O2	168.23	Mono						0.31
128	Sugiol	56.566	C20H28O2	300.4351	phenolic	4.5	4.02	3.74	6.8	6.12	4.49
129	3-O-Methylestradiol	57.284	C19H26O2	286.4085	Diter			0.14			
130	Guaia-1(10),11-diene; α-Bulnesene; δ-Guaiene;	57.47	C15H24	204.3511	sesqui	0.1		0.57		0.16	0.22
131	12-Hydroxyabieta-8,11,13-trien-7-one	61.493	C20H28O2	300.4351	phenolic	0.03		0.49	0.28	0.06	0.21
132	Betulin	70.828	C30H50O2	442.7168	Triter			0.01		0.06	0.06
133	cis-Phytol; trans-Phytol	78.496	C20H40O	296.531	diter	0.03			0.26		0.07
	Total % of Monoterpenes					40.45	39.79	57.08	37.91	48.4	31.85
	Total % of Sesquiterpenes					39.31	30.38	22.95	30.67	25.98	34.64
	Total % of Diterpenes					5.49	0.62	0.5	10.6	8.43	11.34
	Total % of Triterpenes								0.06	0.01	0.06
	Total % of Organic					0.49	0.3	0.42	0.58	0.52	0.94
	Total % of Aromatic					0.26		0.35		0.47	
	Total % of Faty acid					0.15	1.37	0.82	0.19	0.91	0.21
	Total % of Phenolic					4.53	7.08	4.02	6.18	4.23	4.7

**Fig. 4:**
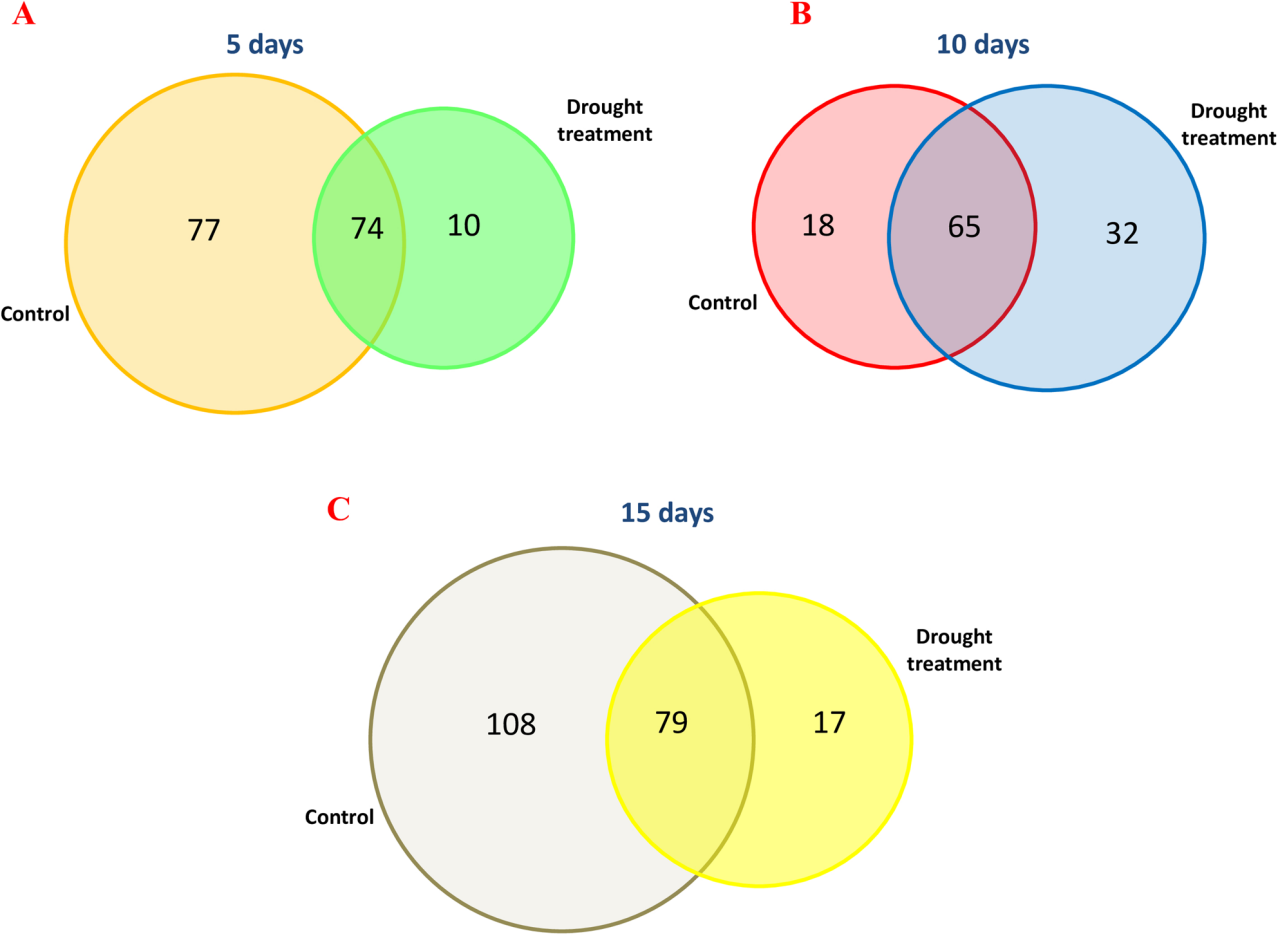
Two-way Venn diagram to show the number of unique and common compounds in the hexane extracts from *S. rosmarinus* plantlet under different drought time. **A** Two-way Venn diagram of the unique and common compounds after 5 days from drought. **B** Two-way Venn diagram of the unique and common compounds after 10 days from drought, **C** Two-way Venn diagram of the unique and common compounds after 15 days from drought

In the context, the six hexane extracts from the different samples under different drought durations and control have unique, common and major compounds see Fig. 4. For instance, the extract from *S. rosmarinus* plantlets at 5DDS and 5DC had 77 compounds unique to control, 10 compounds unique to drought treatment and 74 common compounds shared with the extract from control and drought treatment see Fig. 4A. Beside, the extract from *S. rosmarinus* plantlets at 10DDS and 10DC had 18 compounds unique to control, 32 compounds unique to drought treatment and 65 common compounds shared with the extract from control and drought treatment see Fig. 4B. Also, the extract from *S. rosmarinus* plantlets at 15DDS and 15DC had 108 compounds unique to wild type, 17 compounds unique to drought treatment and 79 common compounds shared with the extract from wild type and drought treatment see Fig. 4C.

Regarding the major terpene compounds, eucalyptol (17.74%) was the major compound in the extracts from *S. rosmarinus* plantlets at 5DC, followed by Caryophyllene (12.77%), Carotol (4.96&), Sclareol (4.88%), D-(+)-Camphor (4.82%), Sugiol (4.5%), Humulene (4.25%), alpha.-Terpineol (4.1%), Elemene isomer (3.57%) and d-Ledol (3.32%). Moreover, eucalyptol (23.14%) was the major compound in the extracts from *S. rosmarinus* plantlets at 10DC, followed by D-(+)-Camphor (9.46%), Humulene (9.03%), Caryophyllene (7.7%), β-Pinene (5.31%), p-menth-1-en-8-yl acetate (4.49%), Sugiol (4.02%), Carotol (2.36%) and alpha.-Terpineol (1.92%). Also, Eucalyptol (20.97%) was characterized as the major compound in the extracts from *S. rosmarinus* plantlets after 15DC, followed by Caryophyllene (8.62%), abd-7,13(E)-dien-15-yl acetate (6.67%), D-(+)-Camphor (6.21%), alpha.-Terpineol (4.69%), Sugiol (3.74%) and Humulene (3.17%) (Table 1, Table S2). On the other hand, we found the major compound in the extracts from *S. rosmarinus* plantlets at 5DDS was Humulene (13.97%), followed by Eucalyptol (12.82%), D-(+)-Camphor (8.83%), Isocaryophyllene (8.08%), Sugiol (6.8%), p-menth-1-en-8-yl acetate (4.4%), β-Pinene (2.86%), Carotol (2.21%), alpha.-Terpineol (1.82%) and Naphthalene, decahydro-, cis- (1.31%). And Eucalyptol (12.45%) was reported as a major compound in the extracts from *S. rosmarinus* plantlets at 10DDS, followed by Sclareol (9.56%), Caryophyllene (8.87%), D-(+)-Camphor (7.37%), Sugiol (6.12%), alpha.-Terpineol (4.73%), Humulene (5.74%), cis-Caryophyllene epoxide (3.22%), L-α-Terpineol (2.62%), p-menth-1-en-8-yl acetate (2.44%), d-Ledol (2.02%), Naphthalene, decahydro-, cis- (1.59%) and (-)-Spathulenol (1.31%). At the end, the Sclareol (11.07%) was reported as a major compound in the extracts from *S. rosmarinus* plantlets at 15DDS, followed by Humulene (8.85%), Caryophyllene (6.94%), Eucalyptol (6.65%), D-(+)-Camphor (5.72%), p-menth-1-en-8-yl acetate (5.37%), Carotol (5.25%), Sugiol (4.49%), trans-Pinocamphone (2.59%), Linolool, formate (2.57%), Elemene isomer (2.09%), Naphthalene, decahydro-, cis- (1.95%), cis-Caryophyllene epoxide (1.51%), alpha.-Terpineol (1.26%), (+)-d-Ledol (1.03%) (Table 1 and S2, and Fig. 2).

In addition, when comparing the type and quantity of terpene compounds in the six samples from the control with and without drought treatments at all times, we found that the level and type of terpenoid compounds that exist at six extracts are varied. So, we suggest that exposure to different periods of drought has an effect on the quality and level of terpenes in the samples. This leads to a very important question: How does exposure to drought over different periods affect the of different levels and types of terpenes? Before we started our work it was difficult to answer this key question due to lack of information at the molecular genetics level, especially drought-related changes in terpene accumulation. So we used the RT-qPCR technique to understand the linkage between drought conditions and terpene accumulation through analysis the expression level of terpenoid and terpene biosynthesis genes under the effect of different drought times.

### Analysis the expression level of terpenoid and terpene biosynthesis genes under the effect of different drought times

**Fig. 5:**
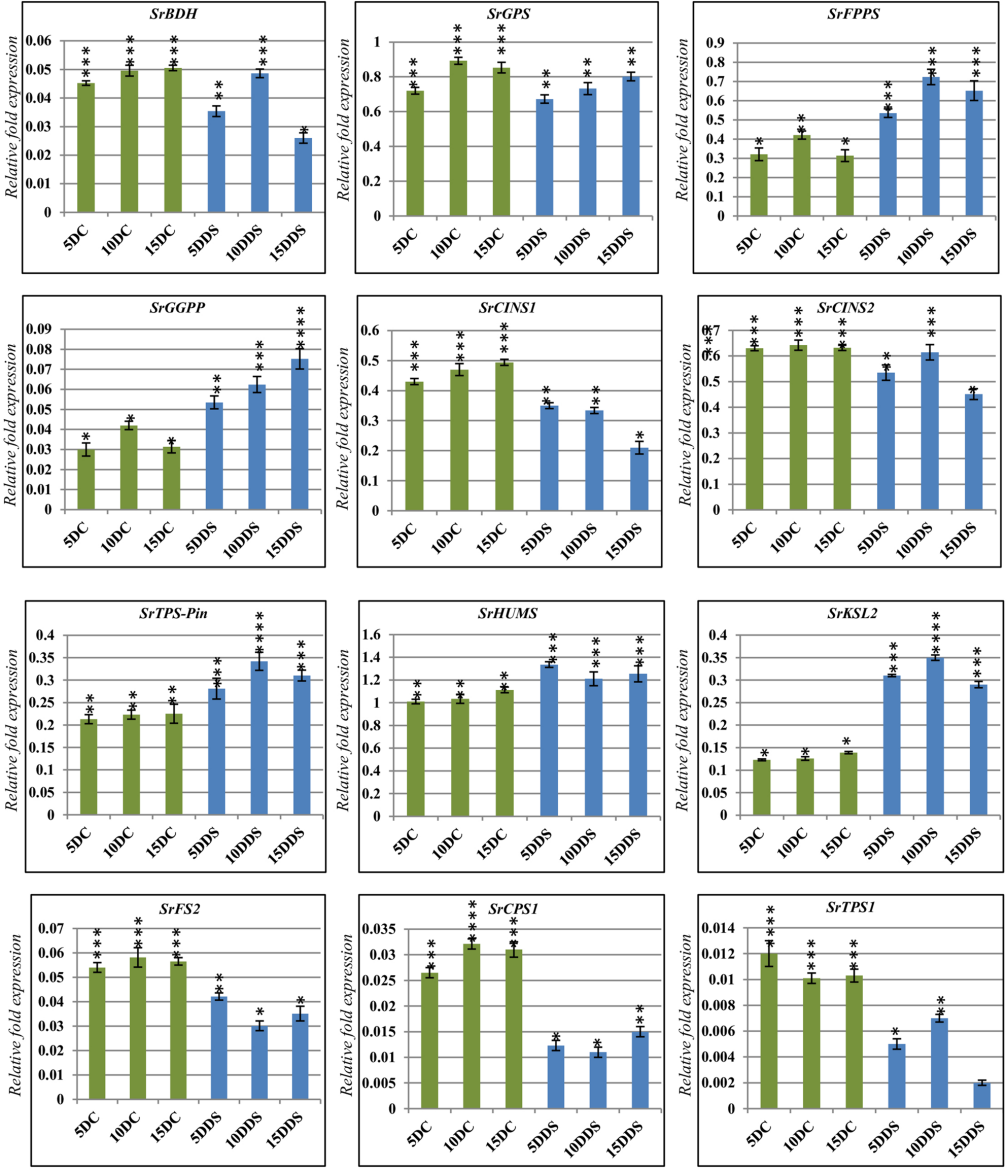
Quantitative Reverse Transcription PCR (RT-qPCR) to validation the expression levels of terpenoid and terpene synthese genes in *S. rosmarinus* plantlet. Total RNAs were extracted from control and treated samples at different times (e.g., 5DC, 10DC, 15DC, 5DDS, 10DDS and 15DDS). The expression of *SrFPPS*, *SrGGPP*, *SrTPS-Pin*, *SrHUMS*, *SrBDH*,* SrGPS*,* SrCINS1*,* SrCINS2*,* SrFS2*,* SrCPS1* and *SrTPS1* genes were analysed using quantitative real-time. *SrACTIN* was used as the internal reference. The values are means ± SE of three biological replicates

To detect the effects of drought at different times on the expression level of terpenoid and terpene biosynthesis genes we select twelve genes which related with the major types of terpenes, and throught analysis the expression levels of these twelve we can understand drought-induced modulation of terpene accumulation. Our results showed that the expression profile of our twelve selected genes at wild and treated samples at different times (e.g., 5DC, 10DC, 15DC, 5DDS, 10DDS and 15DDS) were detected (Fig. 5). For example, the expression of *SrFPPS*,* SrGGPP*,* SrTPS-Pin*,* SrHUMS* and *SrHUMS* genes were upregulated under the effect of drought at all different times (5DDS, 10DDS and 15DDS) in compared with control. While, the expression of *SrBDH*,* SrGPS*,* SrCINS1*,* SrCINS2*,* SrFS2*,* SrCPS1* and *SrTPS1* genes were downregulated under the effect of drought at all different times (5DDS, 10DDS and 15DDS) in compared with wild type (Fig. 4). In the context, we found that all the previous genes have changed in the expression levels at different stress times and under drought and non-drought conditions (control). These results suggest that these previous genes may play key roles in the accumulation of various terpenes in response to drought stress.

### Drought alters various chlorophyll’s contents and the activity of antioxidant enzymes in S. rosmarinus plantlets

**Fig. 6:**
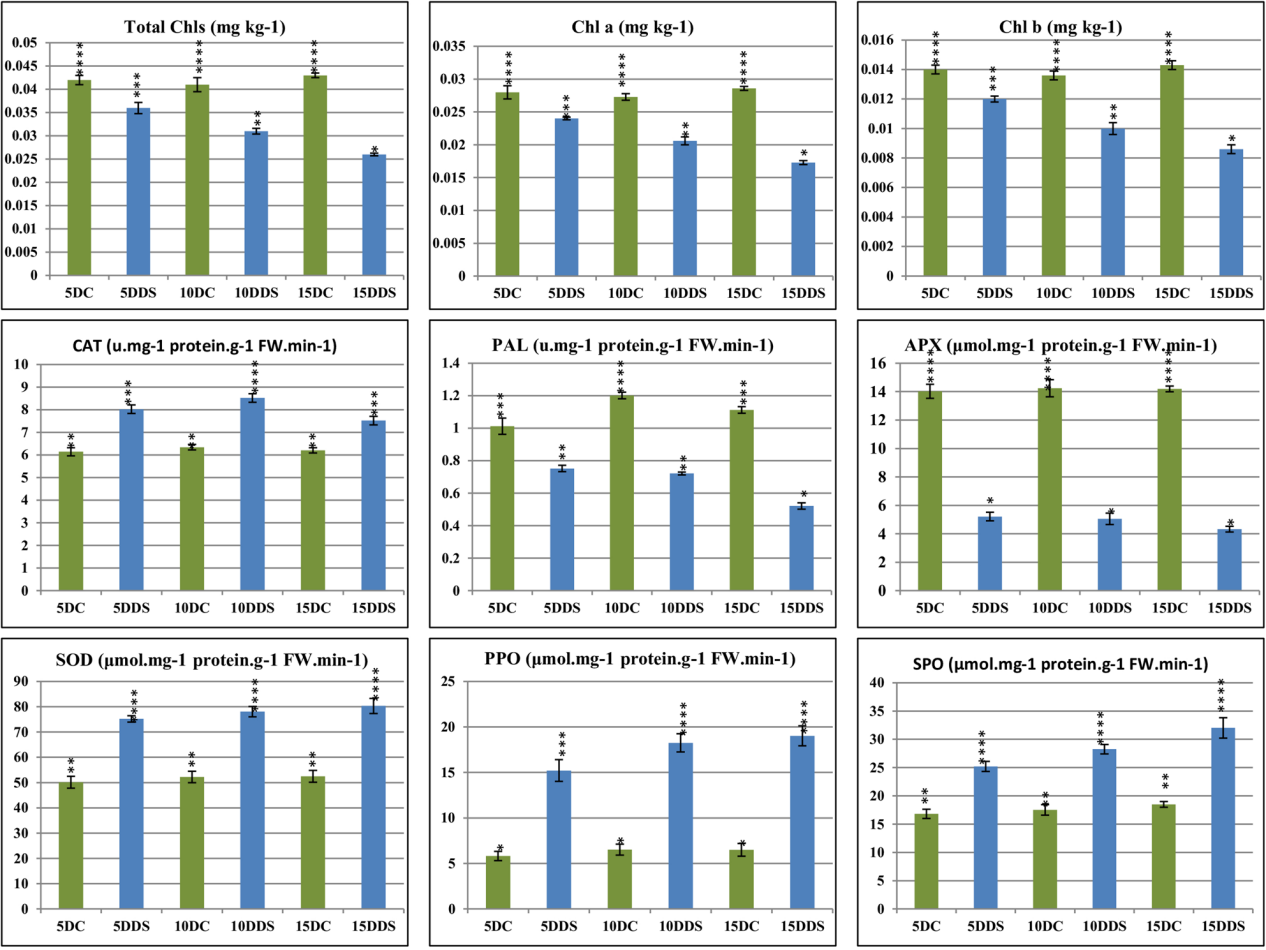
Physiological and biochemical indicators of *S. rosmarinus* plantlet under the effect of different drought time. Analysis of variance (ANOVA) was performed applying, followed by Duncan’s multiple range tests. Significance levels were indicated as (*) for P-values less than 0.05, (**) for *P* < 0.01, (***) for *P* < 0.001, and (****) for *P* < 0.0001, demonstrating the highest degree of significance. This allowed us to determine the effect of drought time that exhibited statistically significant differences in the content of total chlorophyll, chlorophyll a, chlorophyll b and antioxidant enzymes

Chlorophyll’s serve as the primary tools for absorbing light from sunlight then working Through photosynthesis in plants to transfer the energy from light to two forms of energy-storing molecules, which are consumed by plants to convert carbon dioxide and water into glucose [53]. The levels of total chlorophyll, chlorophyll a, and chlorophyll b contents were assessed in *S. rosmarinus* plantlet under various drought durations and control plantlets. Our results indicated a decrease in the levels of these previous components in most of the treatments compared to the control (Fig. 5). Moreover, Fig. 6 shows antioxidant activity data under the effect of various times from drought stressors, and regardless of drought treatment, antioxidant enzymes such as CAT, SOD, PPO and SPO showed higher activities than that of plantlets grown in normal growth conditions (control). However, APX and PAL enzymes significantly exhibited lower activity levels upon exposure to drought stressors at all times in comparison with the control (Fig. 6).

## Discussion

Most plants in the Lamiaceae family in general, and the *Salvia* genus in particular, possess a strong aromatic character due to their diverse types and quantities of terpenes and aromatic compounds, which may reach up to 150 phytochemicals. For this reason, the aerial parts of the plant (stems and leaves) are widely used as flavoring agents in foods, cleaning products, perfumes, aromatherapy, shampoos, and as food preservatives around the world [4, 17–21, 49–51]. Moreover, the growth and biomass of most Lamiaceae plants are affected by various biotic and abiotic stressors, particularly drought [51]. Therefore, in our investigation we studied the growth, biochemical, and genetic changes of *S. rosmarinus* plantlets under different drought durations.

In this study, drought imposed for 5, 10, and 15 days (5DDS, 10DDS, 15DDS) negatively affected the growth of *S. rosmarinus* plantlets, including plantlet size, cell development, and the shape and color of leaves and stems. Similar findings have been reported for rosemary and other Lamiaceae species, where drought and other abiotic stressors negatively impacted growth, metabolic levels, and morphological traits. For instance, water deficit conditions reduced the biomass and growth of *Lavandula latifolia*, *Mentha piperita*, and *Thymus capitatus* [54]. Likewise, significant reductions in fresh biomass were reported in *Salvia miltiorrhiza* and *Ocimum basilicum* under water-deficit stress [55, 56]. Decreased aerial growth of *Mentha spicata*, *Mentha piperita*, and *Rosmarinus rosmarinus* under drought stress has also been documented [57–60]. On the other hand, our findings contradict those of Formica et al. (2024) [37], who reported that drought had no effect on rosemary biomass.

Drought significantly impacts plant tissue size by inhibiting cell division and expansion, primarily due to a loss of turgor pressure. This generally results in an overall reduction in various plant tissue sizes and biomass accumulation [61, 62]. For example, in leaves, which are considered a primary site of drought response, plants develop smaller, thicker leaves with reduced leaf area and shorter leaf length to minimize water loss through transpiration. This is accompanied by anatomical changes in leaves, including an increase in cuticle thickness and the density of mesophyll palisade cells. Bulliform (motor) cells in grasses lose turgor, causing leaves to roll or fold, which further reduces the surface area exposed to sunlight and air [61, 62]. In stems, stem diameter and the thickness of vascular tissues (xylem and phloem) are typically reduced. Lignification (the process of becoming woody) of cell walls around vascular bundles may increase in drought-resistant varieties to improve mechanical strength [63].

We also investigated the effects of drought on the accumulation of various terpenes in the leaves of *S. rosmarinus* plantlets at 5, 10, and 15 days of water withholding, compared with the controls. Terpenoid, aromatic, and other phytochemical compounds were analyzed using GC-MS from six samples (three drought-treated and three controls). At 5DDS and 15DDS, the percentage of total monoterpenes emitted from untreated plantlets was higher than that of drought-treated plantlets, whereas at 10DDS the drought-treated plantlets exhibited higher monoterpene than the wild type. Furthermore, at 5 and 10 days of drought stress (DDS), sesquiterpene levels were higher in untreated plantlets, whereas at 15 DDS, they were elevated in drought-stressed plantlets. Diterpenes and triterpenes were detected only in drought-treated plantlets at all drought durations. The percentages of organic, aromatic, fatty acid, and phenolic compounds varied with drought treatment across the different sampling times. These findings are in line with previous studies [64, 65], which reported increased terpenoid and essential oil contents in rosemary under various drought levels. Other studies also confirmed that controlled mild drought can initially enhance terpenoid levels before decreasing essential oil concentration under severe stress [37, 64–66].

Furthermore, we observed an association between terpene compound production and the expression levels of terpene- and terpene-synthesis-related genes. For example, reduced production of and expression levels for 1,8-Cineole synthase genes were observed in drought-treated *S. rosmarinus* plantlets compared with the wild type.

We also detected associations between total mono-, sesqui-, and diterpene percentages and the expression of genes such as *SrGPS*, *SrFPPS*, and *SrGGPP*. Likewise, relationships were identified between the production of α-pinene, β-pinene, humulene, isocaryophyllene, 9-epi-caryophyllene, caryophyllene, isocaryophyllene oxide, kaur-15-ene, and kaur-16-ene and the expression levels of *SrHUMS* and *SrKSL2*, genes encoding pinene synthase, α-humulene/β-caryophyllene synthase, and kaurene synthase-like 2. The highest levels of these compounds and their gene expression were detected under different drought durations compared with the wild type. These findings agree with several studies reporting association between gene expression and metabolite accumulation, revealing that terpenoid levels can be regulated by transcriptional processes [4, 12, 17, 19, 20, 27, 67–74].

Correspondingly, previous research has shown that drought stress alters the expression of terpene-related genes, with several terpenoid and terpene synthase genes being significantly up- or down-regulated in *Bupleurum chinense*, *Glycyrrhiza glabra*, *Pinus elliottii*, and cumin under drought conditions. Also, these studies confirmed that terpene synthesis-related genes play important roles in hormone responses, protein transport, and secondary metabolism [34, 75–77].

In addition, we evaluated changes in chlorophyll contents and antioxidant enzyme activities in *S. rosmarinus* plantlets. The results revealed clear relationships between metabolic changes and drought duration. Compared to untreated plantlets, drought stress altered total chlorophyll, chlorophyll a, chlorophyll b, and antioxidant enzyme activities (Fig. 5). These findings indicate that chlorophyll content and antioxidant enzyme activities are sensitive to drought duration, subsequently influencing other metabolic pathways. These results agree with previous studies showing that various chlorophyll types decrease with increasing drought intensity in chickpea, blue honeysuckle, and plantain trees [78–81]. Numerous studies also demonstrated significant alterations in antioxidant enzyme activity under drought [82–85]. Similarly, other studies have reported that drought affects chlorophyll levels, antioxidant enzyme activities, terpenoid contents, and the phytochemical profiles of essential oils in *S. rosmarinus*, chickpea, milk thistle, barley, and wheat [82, 86–89].

Several reports have indicated that these effects arise from drought-induced disturbances in physiological and biochemical processes. For instance, drought reduces photosynthesis and alters hormonal balance, particularly by disrupting abscisic acid (ABA) regulation, inducing stomatal closure, and limiting CO₂ entry, thereby impairing energy production [49, 90]. Drought also affects water relations and nutrient uptake, reducing the plant’s ability to absorb and transport water, which ultimately impacts morphological, physiological, and biochemical characteristics [91–94]. Moreover, drought induces oxidative stress by increasing reactive oxygen species (ROS), causing cellular damage and potentially leading to cell death [95, 96].

## Conclusions

Our study was conducted to investigate the effects of drought stress at different time points on growth, chlorophyll content, antioxidant enzyme activity, and terpenoid contents in *S. rosmarinus* plantlets. The accumulation of monoterpenes, sesquiterpenes, diterpenes, triterpenes, organic compounds, aromatic compounds, fatty acids, and phenolics showed an oscillating pattern under drought stress at various time points compared with the control, as drought stimulated different terpenoid backbone biosynthesis pathways to promote their synthesis. Simultaneously, drought stress induced a rapid increase in the activity levels of some antioxidant enzymes, including CAT, SOD, PPO, and SPO, whereas the contents of various chlorophylls and the activity levels of APX and PAL enzymes were decreased.

Our results also indicated that drought significantly impacts plant tissue size by inhibiting cell division and expansion, primarily due to a loss of turgor pressure, generally resulting in an overall reduction in tissue size and biomass accumulation. Moreover, this study provides a systematic overview of the time periods during which rosemary plantlets can tolerate drought stress without experiencing severe damage. Finally, the findings offer new insights into how drought stress affects terpenoid biosynthesis pathways, contributing valuable information that may support the development of drought-tolerant plants through genetic engineering, metabolic engineering, and other biotechnological approaches.

## Supplementary Information

Supplementary Material 1. Table S1: List of Salvia rosmarinus genes and primer pairs used for qRT-PCR.

Supplementary Material 2. Table S2: The list of terpenoid and chemical composition in the hexane extracts of Salvia rosmarinus under different drought times.

## Data Availability

All data generated or analyzed during this study are included in this published article and its supplementary information files.
